# Diagnostic Strategies in the Era of Monkeypox Resurgence: A Comprehensive Analysis

**DOI:** 10.7759/cureus.67154

**Published:** 2024-08-18

**Authors:** Thirumalai Arunagiri, Alagammai Ganesan, Vamsi Ravi Kumaran, Suganandhini Mani, Hemanth Kumar Chanduluru, Chitra Vellapandian, Kanaka Parvathi Kannaiah

**Affiliations:** 1 Pharmacy, SRM College of Pharmacy, SRM Institute of Science and Technology, Kattankulathur, IND; 2 Pharmacy and Pharmacology, SRM College of Pharmacy, SRM Institute of Science and Technology, Kattankulathur, IND

**Keywords:** point-of-care testing, diagnostic methods, clinical symptoms, orthopoxvirus, monkeypox virus

## Abstract

The resurgence of monkeypox (Mpox), an orthopoxvirus infection closely related to smallpox, presents a significant global health challenge. This study presents a comprehensive overview of Mpox, focusing on its clinical manifestations, diagnostic strategies, and testing methodologies. A thorough review of the literature and available data on Mpox, emphasizing diagnostic assays, clinical indicators, and laboratory testing, constitutes the core of this analysis. The study involves insights from Mpox patients and healthcare professionals engaged in its diagnosis and management. Contextualizing the research within the global spread of Mpox addresses the complexities associated with the diagnosis of the disease. The findings illuminate diverse Mpox diagnostic techniques, encompassing viral culture, immunological methods, serology, quantitative polymerase chain reaction (qPCR), electron microscopy, and advanced technologies such as artificial intelligence (AI) and the GeneXpert system. qPCR is highlighted as the benchmark for MPXV detection and quantification. These diagnostic advancements have significantly enhanced the precision and efficiency of Mpox diagnosis, facilitating prompt identification and treatment of infected individuals. The study underscores the critical importance of accurate and timely diagnosis, proper handling and transportation of clinical specimens, and the imperative for point-of-care (POC) testing to control the global spread of Mpox.

## Introduction and background

The reappearance of viral threats is a complicated problem for global public health systems in the shifting landscape of infectious diseases. Among these issues is the re-emergence of monkeypox (Mpox), an orthopoxvirus infection with the potential for cross-species transmission that has sparked renewed interest. The symptoms of this virus range from modest cutaneous signs to serious disease. As we traverse the complexities of managing and preventing viral infections, a thorough examination of Mpox provides useful insights into its various transmission channels, clinical manifestations, and successful preventative techniques. Mpox, which is closely related to the iconic smallpox virus, has numerous modes of transmission, including person-to-person, animal-to-human, and even probable sexual transmission. These various mechanisms of transmission highlight the vital importance of a complete grasp of its epidemiology. As Mpox outbreaks continue to spread across borders, affecting both humans and animals, the importance of robust surveillance systems and widespread public awareness campaigns becomes clearer.

The World Health Organization (WHO) recommends identifying monkeypox by its preferred name, "Mpox" [1]. The orthopoxvirus genus includes the MPXV, which is a member of the Poxviridae family [2]. Mpox is caused by the monkeypox virus (MPXV). This virus has similarities with other orthopoxvirus members, including the cowpox virus, vaccinia virus (which is used to manufacture smallpox vaccinations), and variola virus, the cause of smallpox, which is now eliminated. There are two clades of MPXV, namely the West African and the Congo Basin clades, with mortality rates of 1% and 10%, respectively. The MPXV was initially isolated from a captive Cynomolgus monkey in 1958 at the Statens Serum Institute in Copenhagen, Denmark. Since then, it has been found in many animal reservoirs, mostly in rodents and other small mammal species [3-5]. A pediatric child from the Democratic Republic of the Congo was the subject of the first documented human case in 1970 [6-9]. One hundred sixteen countries reported 91,788 laboratory-confirmed cases of Mpox, including 167 fatalities, from January 1, 2022, to October 31, 2023 [10,11]

## Review

Diagnostic techniques

When determining the cause of viral infections, diagnostic testing is essential. While indirect diagnostic techniques search for the consequences of the infection and direct diagnostic techniques search for the presence of the virus. Cell culture is a fundamental diagnostic technique accomplished in laboratories if infectious viral agent is suspected in patients. The process of growing cells or tissues in a lab is known as cell culture. To promote viral multiplication within the cells, patient samples can be used to infect cell lines and the identification of the virus can be aided by the presence of apparent cytopathic effects (CPE). Immunofluorescence investigations, which employ fluorescently labeled antibodies to detect viral antigens, can also be used to directly identify the virus in patient samples. Virus-specific antibodies to detect viruses in fixed cells or tissues can also make use of infected cells. Enzyme-linked immunosorbent assays (ELISAs), enzyme immunoassays, western blots (WBs), lateral flow immunoassays, and agglutination tests are examples of diagnostic immunoassays. Based on the concept of polymerase chain reaction (PCR) or nucleic acid hybridization, assays for detecting viral nucleic acids are incredibly sensitive and specific for a particular virus [2].

Clinical symptoms

**Figure 1:**
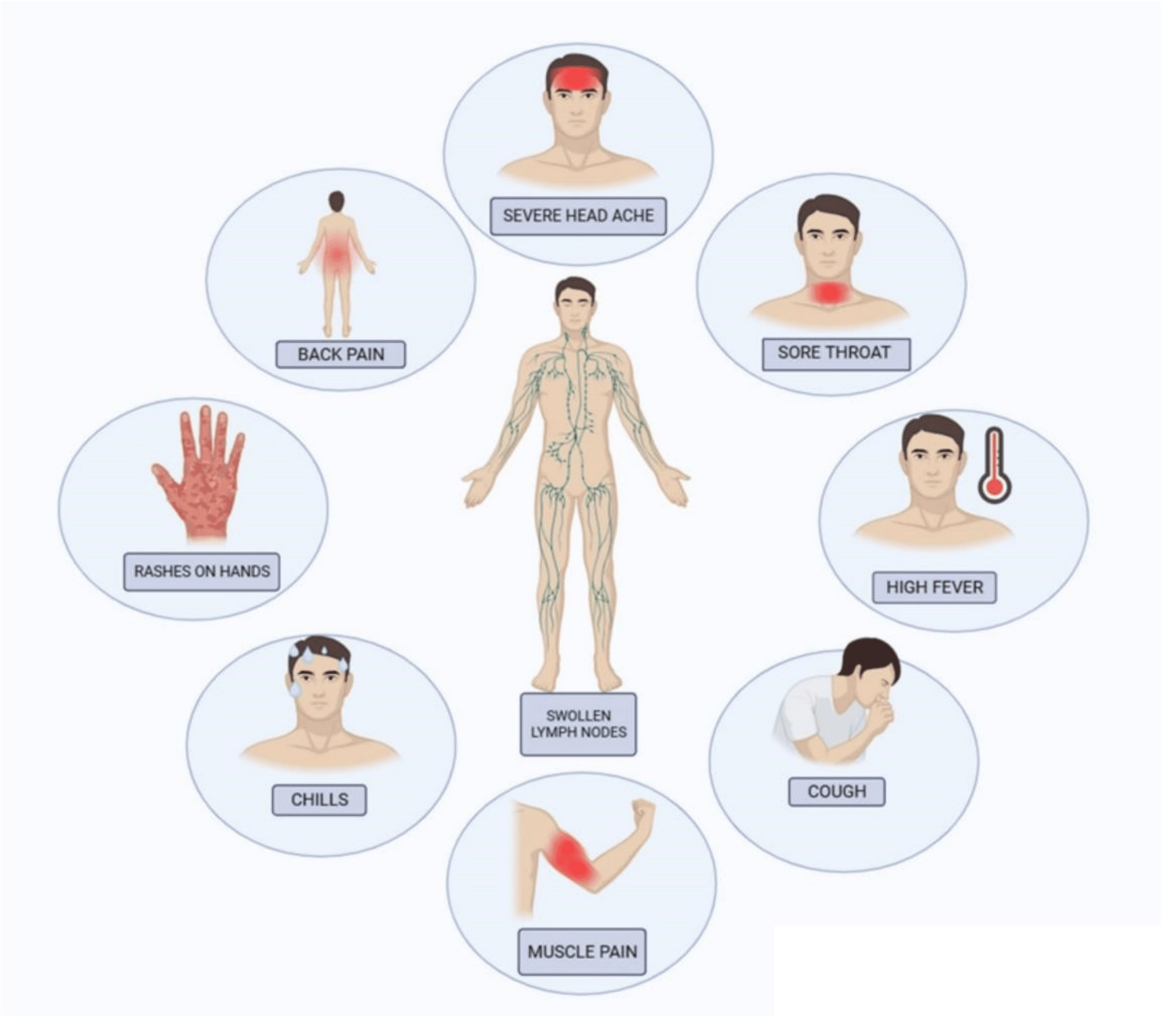
Various clinical symptoms of Mpox disease in human Created a sketch using BioRender. Mpox: monkeypox

The symptoms of Mpox include high temperature (fever), chills, headache, enlarged lymph nodes, muscle aches, and a painful cutaneous eruption that usually starts as raised bumps on the skin and spreads to the face, genitalia, and extremities as picturized in Figure 1 [12,13]. However, it shares symptoms with other viral illnesses such as enterovirus, molluscum contagiosum virus, varicella-zoster virus, and herpes simplex virus infections [14-16]. Since the symptoms of Mpox are similar to those of other viral infections, determining the virus based on its clinical symptoms is challenging; hence, laboratory testing is required.

Laboratory testing

To establish the possibility of infection, a test must be performed using clinical and epidemiological parameters. Testing is limited not only by money and equipment but also by the proper means of sample collection and highly qualified workers [17,18].

Specimen collection

**Figure 2:**
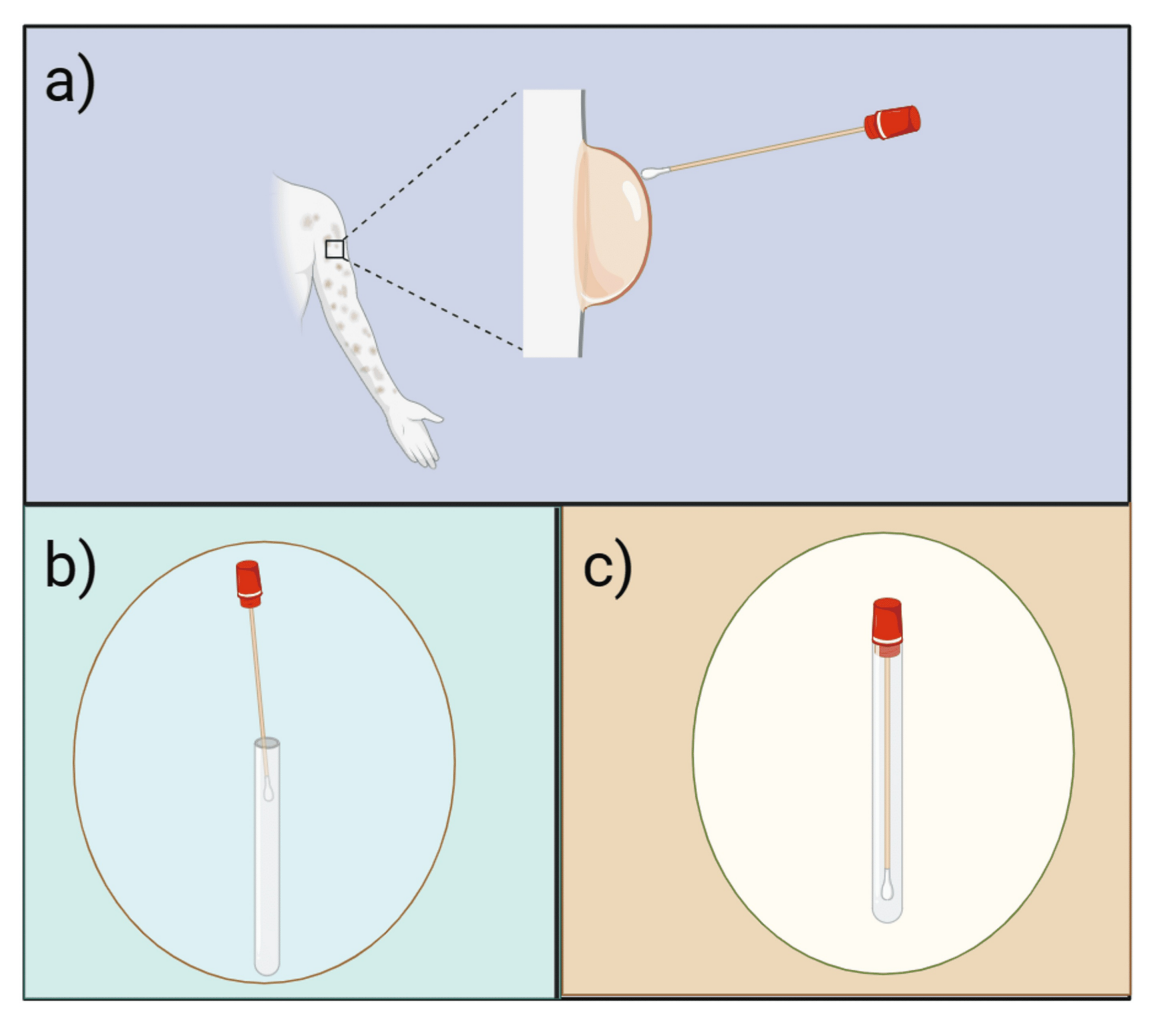
It shows the sampling method; two to three lesions should be sampled by utilizing two swabs per lesion to collect adequate DNA. (a) Depicts the collection of samples from the lesion. (b) Depicts the break of the swab applicator into the sterile, sealed tube using the molded breakpoint. (c) Depicts the transport of the dry swab in a tube using a viral transport medium under required conditions with the following of proper guidelines according to their laboratories Created a sketch using BioRender.

MPXV can be identified by skin lesions, anal, rectal, and oropharyngeal swabs; however, because there is a deficit of clinical evidence about the use of oropharyngeal specimens in Mpox diagnosis, the results of these specimens should be interpreted cautiously. With approval from the ethical review committee, and provided that suitable laboratory and medical competence is available to collect, transport, and preserve the specimens, urine, sperm, and rectal or vaginal swabs may also be included depending on clinical markers such as the location of the lesion. [19-21]. The diagnosis of MPXV may be aided by testing ethylenediaminetetraacetic acid (EDTA)-anticoagulated whole blood; however, as viremia is only detected early in the course of the disease, before the prodromal phase with a skin lesion, the sample may not contain substantial levels of virus. Medical personnel should utilize sufficient personal protective equipment (PPE) and collect samples according to approved standard operating procedures (SOPs) [21]. The sampling method of Mpox lesions for diagnosis is clearly depicted in Figure 2.

Packaging and shipment of clinical specimens

Appropriate handling and storage of specimens during transportation is necessary for accurate testing and diagnosis. Clinical specimens, viral isolates, and cultures from suspected, probable, or confirmed Mpox cases should be sent as Category A, UN2814 "infectious substances affecting humans" while traveling abroad. According to Section 1.5.1 of adverse drug reaction (ADR), certain countries have chosen to reclassify MPXV to UN3373 Category B [22]. This affects MPXV, which is transported by road under the multilateral agreement M347 [23]. All specimens being transported should be properly triple-packaged, labeled, and documented. For further information on the shipping requirements for infectious substances in 2021-2022, please refer to the WHO guidance on regulations [19,20].

Storage

Specimens must be frozen at -20°C or below or chilled to 2-8°C within an hour after collection, and they must be sent as quickly as possible to the testing facility. Completing accurate diagnostic tests requires proper sample handling and storage during travel. Samples that have been collected more than 60 days ago should be kept for extended periods at -70°C. The above-mentioned storage procedures are essential for preventing deceptive adverse effects. Poor specimen quality, improper handling or transportation, or assay-related technical problems (such as an unsuccessful DNA extraction) might have all affected the reference laboratory's ability to execute diagnostic tests and maintain quality control [19-21].

Conventional methods of diagnosis

**Figure 3:**
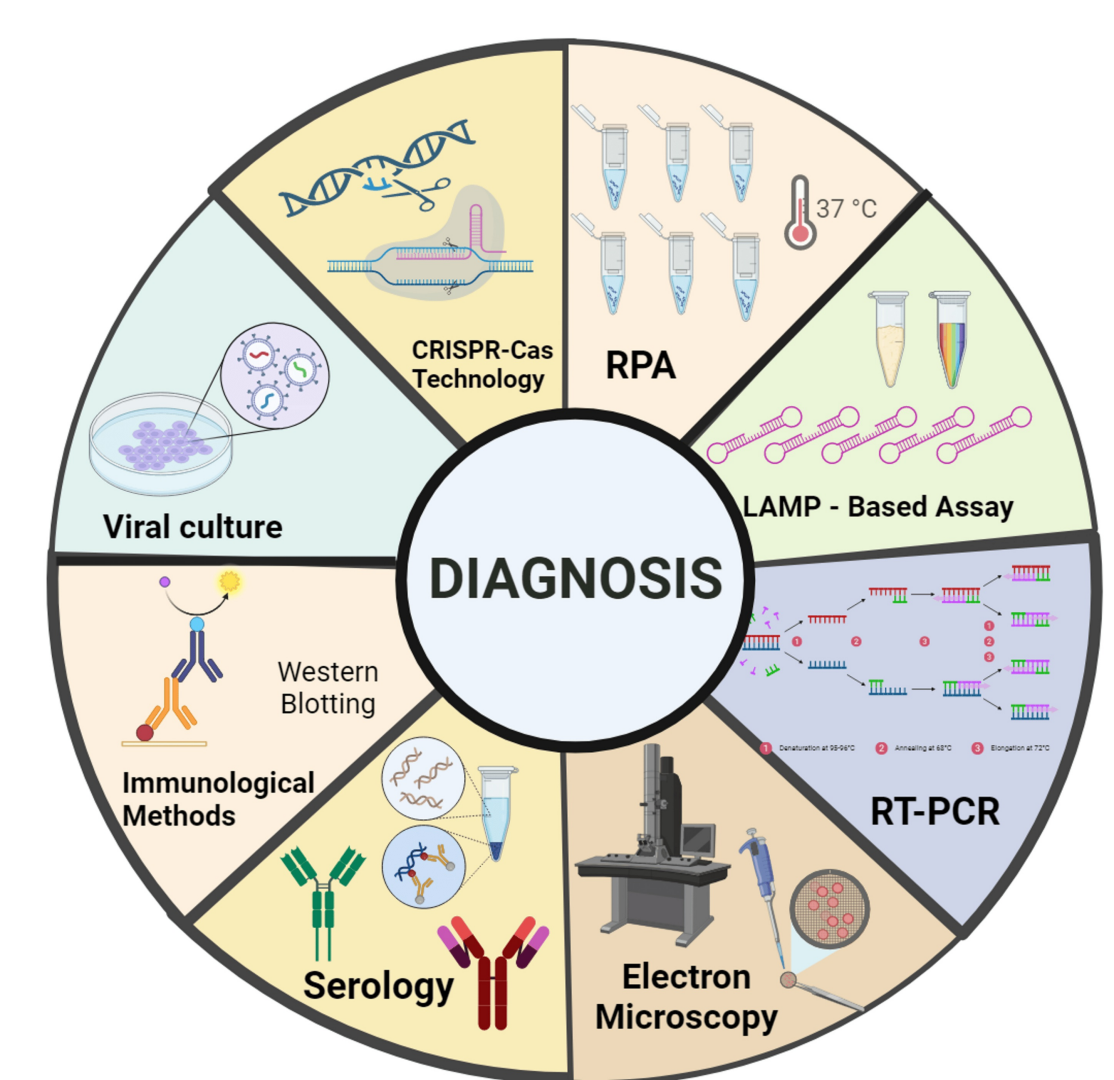
This shows the various diagnostic techniques of Mpox disease Created a sketch using BioRender. Mpox: monkeypox

The typical methods of MPXV detection that have been employed in the past decades include electron microscopy, serology, viral culture, and immunological approaches. These methods are pictorially illustrated in Figure 3.

Viral culture

Human amnion cells and monkey kidney (HeLa) cells are two examples of cell lines that may be used to produce MPXV and were used in the process of viral isolation. Infecting immortalized cell lines such as HEP-2 (human epithelial carcinoma cells), Vero (African green monkey kidney cells), and pig embryonic kidney (PEK) cells are allowed for the isolation [24]. Additionally, Vero, Vero E6, Vero 76, BSC-1, HEP-2, PEK, MA-104, HeLa, BSC-40, LLC-MK2, and Balb/3T3 clone A31 cell lines can be used to cultivate MPXV [6]. It is recommended to carry out the MPXV culture process in a high-containment laboratory (PC3/BSL3), and the laboratory staff doing this operation should be vaccinated. Cells may be harmed by swabs that include semisolid medium or additives (such as gel or charcoal) [24]. In short, the CPE demonstrates the cellular infectivity of the isolated virus. Worldwide, public health and research laboratories have employed culture-based approaches for MPXV detection; however, because virus isolation is challenging, insensitive, and necessitates BSL-3 equipment, the WHO does not formally support it as a standard diagnostic procedure [6].

In one of the initial studies, Lapa and colleagues detailed the isolation of MPXV from a semen specimen obtained early in the course of the disease from a patient with prolonged seminal viral shedding. The researchers used sperm collected on the sixth day after the beginning of symptoms to infect Vero E6 cells to isolate the virus. Forty-eight hours following inoculation, a pronounced cytopathic impact was seen, and real-time PCR confirmed MPXV replication. Comparably, a recent study employed Vero E6 cells to separate live MPXV from urethral and anal swabs [20,25].

Immunological approaches

The immunological detection methods for MPXV include immunohistochemistry (IHC), WB, radioimmunoassay (RIA), and ELISA [26]. IHC determines the quantity, distribution, and localization of a target inside a tissue [27]. The concepts behind both IHC and ELISA are similar: the former looks for viral antigens in tissues or cells, while the latter looks for IgG and IgM antibodies [28]. IHC tests are being performed to seek out orthopoxvirus-specific antigens. Blood samples are taken for testing and the presence of antigens indicates that the individual is susceptible to Mpox viruses [29]. The MPXV A29 protein is the virus's envelope protein, which enables virus identification by host cells and is the most important protein target for MPXV immunoassays [28]. ELISA is the recommended approach for detecting serum antibodies; specific IgM and IgG antibodies can be found in infected patients in seven to 21 days after the development of a rash [30]. However, smallpox immunization may affect ELISA results [31]. A quick test kit for lateral flow immunoassay cassette-based MPXV antigen and antibody detection during infection has been developed by JOYSBIO with great success [19]. One benefit of this fast detection kit is that it can produce findings in 15 minutes or less. The kit is incredibly easy to use compared to earlier detection techniques, and medical staff may gather test specimens from patients' skin lesions with ease.

Serology

Serological testing for Mpox infection is beneficial in a variety of circumstances, including identifying self-attenuated infection, determining asymptomatic illness, and monitoring immunity. However, because of the paucity of commercial assays, Mpox serology is not widely used in diagnostic laboratories [32,33]. IgM antibodies can be detected five days after infection using an enzyme-linked immunosorbent test, whereas IgG antibodies can be detected eight days later using an ELISA. However, because MPXV and other orthopoxviruses exhibit antigenic cross-reactivity, the specificity is inadequate [28,33]. There is a four-fold increase in antibodies that aid in diagnosis in both the acute and convalescent periods [33].

Quantitative polymerase chain reaction:

For MPXV, quantitative PCR (qPCR)-based molecular diagnostic tests are often employed, while several techniques based on loop-mediated isothermal amplification (LAMP) are currently being investigated. Primers and probes for these assays are designed using conserved regions of the inverted terminal repeats (ITS) region of the virus and the central coding area. For the detection of MPXV or orthopoxvirus, commercial reagent kits with comparable sensitivity and specificity are available. Disseminated VACV may be distinguished from spontaneous infection by multiplexing the detection of MPXV and orthopoxvirus. Multiplexed PCR and LAMP formats are used in clade-specific testing to identify MPXV genomic regions that differ slightly in nucleotide composition between Clades I, IIa, and IIb. However, in Mpox-endemic countries, there is a testing gap since there are not enough rapid molecular point-of-care tests (POCT) [34-37].

Electron microscopy

Electron microscopy can be used to detect viral components in a scab material, vesicular fluid, biopsy specimen, or viral culture, which can also differentiate between an orthopoxvirus and a herpes virus [38]. Transmission electron microscopy (TEM) was originally applied during a smallpox outbreak in the US in 1947 to distinguish between herpesvirus and poxvirus infections. Despite the use of contemporary TEM techniques and apparatus, it is feasible to verify MPXV infection from high-titer materials, such as vesicle fluids, in less than 30 minutes [39]. Mature extracellular viruses within keratin fibers were evident in TEM pictures, together with the outer membrane. Among the virions seen assembling within vesicles in keratinocyte cytoplasm, a few mature internal viruses resembled bricks, while others possessed an outer membrane with superficial tubules and a biconcave-shaped inner core encircled by lateral bodies. Virion immaturity is currently developing. Particles that were round and lacked a visible center emerged. Clusters of protein particles were also discovered. The intracytoplasmic virus did not change in maturity, but the cytoplasmic viral particles did. The breath varied from 130 nm to 273 nm (median size: 198.38 nm, standard deviations: 6 25.71), while the length varied from 186 nm to 98 nm (median size: 277.6 nm; standard deviations: 6 38.65) [28,29].

Nucleic acid amplification test

The gold standard diagnostic method for detecting and quantifying the MPXV is qPCR [40]. Before COVID-19, PCR had limited use due to equipment and knowledge requirements [41]. By amplifying the envelope protein (B6R) and DNA polymerase (E9L) genes, Li et al. (2006) developed qPCR [42]. It assesses viral load, shedding, and illness progression [41]. A number of generic real-time PCR tests for MPXV have been developed to distinguish the virus from other orthopoxviruses, either directly or by using melting curves to distinguish various orthopoxviruses following PCR amplifications [43,44]. To distinguish between the MPXV strains (i.e., Clade I and Clade II), DNA sequencing is required following PCR amplification [45]. Chemagic MSMI (PerkinElmer) was used to extract specimens, and QuantStudio 12 K Flex (ThermoFisher) or Cobas 6800 (Roche) was used to amplify them. Primers created in the lab targets conserved viral polymerase gene areas shared by the orthopoxvirus genus. Tumor necrosis factor (TNF) receptor gene differences between MPXV clades and orthopoxviruses are important for strain identification [41]. Mpox strains were identified using PCR techniques that targeted TNF receptor and complement binding protein (C3L) genes. The hemagglutinin (HA) gene identifies pan-orthopoxvirus species; however, species identification requires sequencing [42].

Modern methods of diagnosis

The modern diagnostic methods of Mpox was discussed in detail in Table 1 with their application and impact along with the examples and some of the methods was illustrated in Figure 3.

**Table 1:** Emerging diagnostic technologies in Mpox LAMP: loop-mediated isothermal amplification; RPA: recombinase polymerase amplification; AI: artificial intelligence; POC: point-of-care; Mpox: monkeypox; CRISPR-Cas: clustered regularly interspaced short palindromic repeats-associated proteins; MASTR: Mpox at-home Self-Test and point-of-care

Technology	Application and impact	Examples and explanation
LAMP-based assays	Rapid and simple nucleic acid amplification for diagnosis	The LAMP assays provide quick and accessible detection of Mpox DNA in resource-limited settings
CRISPR-Cas Technology	Precise gene editing and diagnostic capabilities	CRISPR-Cas technology offers accurate detection of Mpox DNA and the potential for targeted therapies
RPA	Isothermal amplification in field conditions	RPA enables fast and specific detection of Mpox DNA in remote areas without the need for complex equipment
GeneXpert system	POC diagnostic platform for rapid results	GeneXpert's portable system facilitates quick and reliable detection of Mpox DNA, enhancing field diagnostics
AI	Automated image recognition and analysis	AI aids in diagnosing Mpox through automated image analysis, supporting healthcare professionals with accurate assessments
MASTR Pouch	Self-testing and quick analysis of viral particles	The MASTR Pouch incorporates RPA and CRISPR for fast, self-testing of Mpox viral particles, enhancing global detection efforts
Dot immunoassay	Sensitive, quick, simple, and low-cost test	The dot immunoassay detects orthopoxvirus in crude and clinical samples within 35 minutes, suitable for BSL 3 biocontrol protocol research and effective in various biosecurity applications

Point-of-care

An ideal point-of-care (POC) diagnostic system should be inexpensive, sensitive, specific, readily available for non-trained individuals to use, quick and reliable, devoid of complex equipment, and accessible to individuals who require it. POC testing of MPXV involves the detection of nucleic acids, antigens, and antibodies. Two molecular POC tests have received emergency use authorization (EUA) from the FDA. One detects DNA from MPXV (clade II only) and orthopoxvirus in specimens, which is collected from the human lesion, while the other detects DNA from MPXV (clades I and II) in human lesion swab specimens. Both tests have been validated using FDA-cleared real-time PCR tests. Independent clinical evaluation of POC molecular assays is underway, with results expected in Q1 2024. The WHO encourages further research to determine the diagnostic accuracy and utility of these critical tools in settings where MPXV clades I and/or II circulate [11].

XpertMpox test

**Figure 4:**
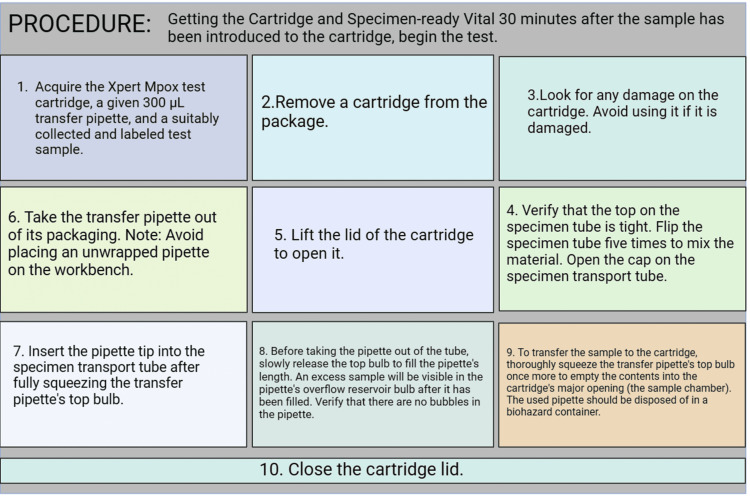
The flow chart represents the procedure involved in the diagnosis of Mpox using the XpertMpox test kit

An automated in vitro diagnostic test called the XpertMpox test is used to qualitatively identify and detect non-variola orthopoxvirus DNA and MPXV clade II DNA. It is demonstrated on GeneXpert® Instrument Systems, which immortalized sample preparation, amplification, nucleic acid extraction, and determining using qPCR tests. The system includes reagents for detecting MPXV-clade II and orthopoxvirus targets in lesion swab specimens. To verify correct sample processing and functioning reagents, the test involves sample processing control (SPC), sample adequacy control (SAC), and probe check control (PCC). The XpertMpox test is designed to be paired with lesion swabs obtained from healthcare providers from patients suspected of having Mpox. The procedure of XpertMpox test is clearly explained in Figure 4.

## Conclusions

Mpox, an orthopoxvirus infection, is a severe danger to global public health due to its potential for cross-species transmission. Highlighting the importance of in-depth knowledge of its diagnostic techniques for valid testing results, proper specimen collection, handling, and transportation are required. In this article, diagnostic methods of Mpox are typically described as conventional and modern techniques. MPXV detection methods that have been used in recent decades are represented as conventional methods, which include viral culture, immunological approaches, serology, and electron microscopy. Immunological methods detect self-attenuated infection, asymptomatic illness, and immunity. Cell lines were used to infect viral cultures. The qPCR technique is commonly employed. It additionally looks into emerging modern methods for diagnosis that are faster, more specific, accurate, and non-time consuming, such as LAMP assays, CRISPR (clustered regularly interspaced short palindromic repeats) technology, and RPA assays. It also explores the XpertMpox test, an automated in-vitro diagnostic test. Using modern TEM procedures and equipment, it is possible to confirm MPXV infection from high-titer samples, such as vesicle fluids, in less than 30 minutes. GeneXpert is the other approach that can be utilized within less advanced laboratories and field settings to enable efficient management and surveillance for Mpox infection. Artificial Intelligence (AI) in the diagnosis of Mpox infection is clinically utilized by an expert analysis of the distinctive skin lesions in the absence of laboratory PCR testing. Many studies on the use of AI to aid in real-time picture detection of Mpox skin lesions have been reported. The MASTR Pouch (Mpox at-home Self-Test and point-of-care Pouch) provides immediate results and is easy to use, enabling routine Mpox self-testing. When contrasted with both traditional and modern values, anything old is valued as gold. As a result, during outbreaks, qPCR is employed as a gold standard test for detecting and quantifying MPXV. However, further investigation into modern approaches such as the XpertMpox test and MASTR Pouch for diagnosing Mpox disease should be endorsed, as these methods are faster, more specific, accurate, and less time-consuming.
